# 89. Follow-Up Blood Cultures (FUBC) in the Management of Gram-Negative Bacilli (GNB) Bloodstream Infections (BSIs): Frequently Obtained and Rarely Helpful

**DOI:** 10.1093/ofid/ofab466.291

**Published:** 2021-12-04

**Authors:** Evan D Robinson, Heather Cox, Amy J Mathers

**Affiliations:** 1 University of Virginia, Charlottesville, Virginia; 2 University of Virginia Health System, Charlottesville, VA

## Abstract

**Background:**

While GNB BSIs remain a major cause of morbidity and mortality, no clear guidelines exist on the utilization of FUBCs to guide management. Despite the recognition of persistent bacteremia as a risk factor for increased mortality, early studies suggested FUBCs were low yield in this setting, and thus had low utility. More recently, some controversy has arisen with multivariate analyses suggesting FUBC acquisition may be associated with lower mortality. We sought to characterize the utilization and yield of FUBCs for GNB BSIs at our institution.

**Methods:**

We performed a retrospective review of 514 episodes of consecutive blood cultures from unique adult inpatients with GNB BSI between July 2017-July 2019. Exclusion criteria included prior positive culture, polymicrobial Gram stain, or discharge, death, or comfort measures only within 24 hours of Gram stain. FUBCs were defined as blood cultures collected between 24 hours to 7 days after the index blood culture. Baseline clinical and microbiologic characteristics were compared between groups, as well as clinical outcomes.

**Results:**

Of 514 episodes, 338 (66%) had FUBCs performed, with a median of 2 FUBCs/episode. The majority of FUBCs (322/338; 95%) were negative, with 9 (3%) yielding the same organism and 9 (3%) yielding a different organism. Most initial FUBCs were obtained prior to index antimicrobial susceptibility results (227/338; 67%). Patients with FUBCs performed had a higher median Pitt bacteremia score (2 vs 1; p = 0.015) and were more likely to have hospital onset (36% vs 22%; p = 0.002), severe neutropenia (16% vs 4%; p < 0.001) and a catheter-associated source (13% vs 4%; p = 0.001). 30-day mortality did not differ between patients with or without FUBCs (10% vs 11%; p = 0.84).

**Conclusion:**

FUBCs were frequently obtained, but were of low yield even in comparison to recent similar studies. Though FUBCs were performed in more severe cases, a difference in mortality was not observed. Delaying the decision of whether to obtain FUBCs until after index antimicrobial susceptibility results are available would reduce unnecessary testing in most cases. Further study could better define where FUBCs after antimicrobial susceptibility testing would be most helpful.

**Disclosures:**

**All Authors**: No reported disclosures

